# The Pharmacology of Xenobiotics after Intracerebro Spinal Fluid Administration: Implications for the Treatment of Brain Tumors

**DOI:** 10.3390/ijms22031281

**Published:** 2021-01-28

**Authors:** Justine Paris, Eurydice Angeli, Guilhem Bousquet

**Affiliations:** 1 Institut National de la Santé Et de la Recherche Médicale (INSERM), U942, 9 Rue de Chablis, 93000 Bobigny, France; justinesv.paris@gmail.com (J.P.); eurydice.angeli@gmail.com (E.A.); 2 Assistance Publique Hôpitaux de Paris, Avicenne Hospital, Department of Medical Oncology, 93000 Bobigny, France; 3 Sorbonne Paris Nord University, 99 Avenue Jean Baptiste Clément, 93430 Villetaneuse, France

**Keywords:** brain metastases, blood–brain barrier, intrathecal injection, glymphatic system, efflux receptor, neonatal Fc receptor

## Abstract

The incidence of brain metastasis has been increasing for 10 years, with poor prognosis, unlike the improvement in survival for extracranial tumor localizations. Since recent advances in molecular biology and the development of specific molecular targets, knowledge of the brain distribution of drugs has become a pharmaceutical challenge. Most anticancer drugs fail to cross the blood–brain barrier. In order to get around this problem and penetrate the brain parenchyma, the use of intrathecal administration has been developed, but the mechanisms governing drug distribution from the cerebrospinal fluid to the brain parenchyma are poorly understood. Thus, in this review we discuss the pharmacokinetics of drugs after intrathecal administration, their penetration of the brain parenchyma and the different systems causing their efflux from the brain to the blood.

## 1. Introduction

Since 1914 and the development of salvarsan to treat syphilis and its neurological damage, penetration of drugs into the brain has continued to be a pharmaceutical challenge [1]. The central nervous system is a sanctuary site, highly protected from the outside environment by the low permeability of the blood–brain barrier (BBB) and by efflux systems preventing brain penetration and diffusion of most xenobiotics [2]. Since recent advances in molecular biology and the development of specific molecular targets, knowledge of the brain distribution of drugs has become a pharmaceutical challenge.

Among the different cancer types, the incidence of brain metastases ranges from 9% to 30% across studies [3,4,5,6,7]. Melanoma, breast and lung cancers are the main providers of brain metastases which occur in 6 to 56% of patients [3], and lead to short survival of under a year [8]. To date, standard treatments are more or less limited to surgery and radiotherapy [9,10].

Systemic anticancer treatments, including the classic chemotherapies and targeted treatments, have a limited benefit on cancer brain metastases because they do not readily cross the BBB, thus obtaining only insufficient pharmacological concentrations in the brain [2].

To overcome this problem, direct intrathecal injections of drugs have been proposed for the treatment of carcinomatosis meningitidis and parenchymal brain metastases [11,12]. However, little is known about drug pharmacokinetics in the brain parenchyma after intracerebrospinal fluid administration.

In this review, we provide a summary of knowledge on drug penetration into the brain parenchyma after intracerebrospinal fluid injection and the different systems causing their efflux.

## 2. Turnover in the Glymphatic System

Brain extracellular spaces occupy 20% of the total brain volume [13], including fluid compartments with blood vessels, cerebrospinal fluid (CSF) and intracellular fluid.

On the cortical surface of the brain, the cerebral arteries extend into the pial arteries running through the subarachnoid and subpial spaces. When they penetrate the brain parenchyma, the pial arteries create a perivascular space filled with CSF and bordered by leptomeningeal cells, known as the Virchow–Robin space. As the pial arterioles penetrate the deeper brain parenchyma, the Virchow–Robin spaces become continuous with the basal lamina, which separate endothelial cells, pericytes and astrocytes. Unlike peripheral tissues, the central nervous system completely lacks lymphatic vessels [14]. 

Since 1981, various experiments have been carried out by H. F. Cserr’s team to assess the clearance of physiological molecules and xenobiotics after their intracerebral administration. Despite major differences in molecular sizes and diffusion coefficients, efflux rates are roughly similar, whatever the molecule considered, suggesting a convective system of brain circulation and efflux, independent from the blood–brain barrier [15].

**Figure 1:**
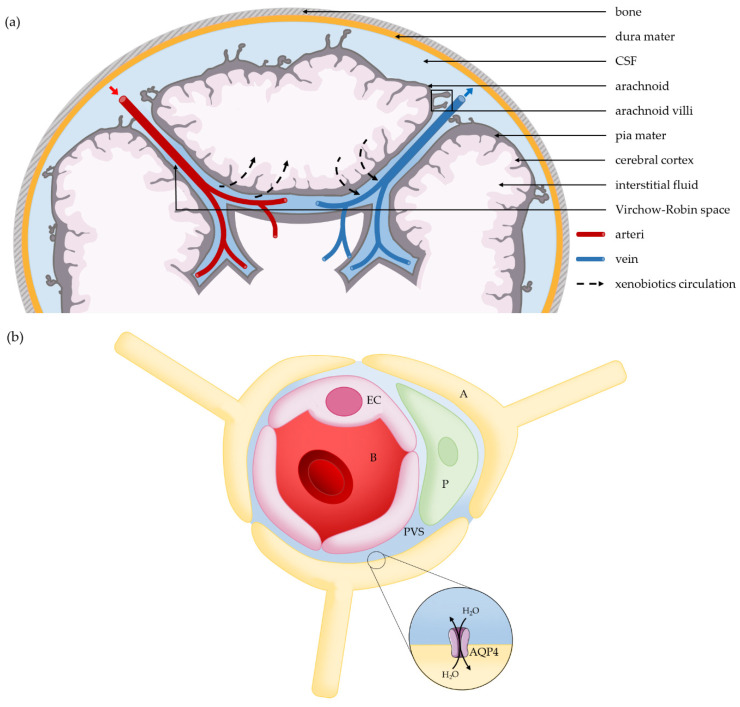
Anatomical structures of brain vasculature. (**a**) The glymphatic system: xenobiotics circulation, diffusion and efflux along perivascular spaces after intrathecal administration. (**b**) Cross section of a brain vessel: xenobiotics circulate along perivascular spaces of blood vessels. A: astrocyte, regulator of the hydrostatic pressure by water exchanges; AQP4: aquaporin 4; P: pericyte; EC: endothelial cell; PVS: perivascular space; B: blood. (Images modified from © SMART/CC-BY-3.0).

This system, called the glymphatic system, is driven by pressure gradients along arterial and venous perivascular spaces [16] (Figure 1a,b), and has an important role in the distribution and clearance of molecules and toxins. Radio-labelled tracer studies indicate that 40 to 80% of large proteins and solutes are removed from the brain by the glymphatic clearance system [13]. 

### 2.1. Transport through the Glymphatic System, Astrocyte Regulation Function

Illif et al. [17] studied the mouse brain distribution of fluorescent and radio-labelled tracers after intrathecal administration. First, they evidenced an absence of brain penetration when drugs were injected into the ventricles, while global brain penetration varied from 20 to 95% after injections into the cisterna magna. Low molecular weight molecules (TR-d3, 3kD) penetrate the brain interstitial fluid from perivascular spaces and the pial surface, while larger molecular weight molecules (FITC-d2000, 2000 kD) remain in the perivascular spaces of penetrating arteries and do not reach the interstitial fluid. Using two photon laser scanning microscopy, fluorescent dextran was able to penetrate brain parenchyma after injection into the cisterna magna and confirmed the passage of small molecules (TR-d3) into the brain interstitium, while larger molecules (FITC-d2000) remained confined to the paravascular spaces of penetrating arteries. After injection, the smaller tracers rapidly entered the brain via surface cortical arteries, in the space surrounding the arterial smooth muscle cells. They then reached the penetrating arterioles and the terminal capillary bed, circulating in a space bounded by astrocytic endfeet. Finally, fluorescent tracers rapidly reached deeper brain structures like the basal ganglia and the thalamus. 

Perivascular astrocytes, which almost entirely cover the brain microvasculature [18], play a major role in CSF and brain interstitial fluid distribution and bulk flow. First, astrocytic endfeet may have a sieving function, allowing the passage of molecules smaller than 20 nm into the brain interstitium, corresponding to the space between two overlapping endfeet [18]. Secondly, they regulate the flow of water by the expression of aquaporin 4 at their endfeet. A knockout mouse model for aquaporin 4 showed a cessation of tracer movements from the distal paravascular space to the interstitium, and of interstitial bulk clearance from the brain parenchyma [17,19]. By creating an arterio-venous hydrostatic pressure gradient, aquaporin 4 facilitates the influx of subarachnoid CSF from para-arterial spaces into the brain interstitium and the clearance of brain interstitial fluid to the extracerebral area. 

Other factors are responsible for hydrostatic pressure modifications in the glymphatic system and the accumulation of toxins. One study demonstrated a reduction of 80–90% for glymphatic flux in elderly mice compared to young mice, leading to an accumulation of tau and amyloid β peptide [20]. This can be due to a reduction of arterial pulsatility, because of the stiffening of the arterial wall in elderly people [21,22], or a reduction of CSF production [21,23,24]. In contrast, a sleeping state (anesthetized or naturally sleeping) significantly improves the performance of glymphatic activity, with a 90% higher CSF flow in the waking state as was demonstrated in rats [25].

### 2.2. Drug Diffusion in the Extracellular Space of Brain

Drug diffusion in the extracellular space responds to diffusion mechanisms, driven by the kinetic energy of molecules and their collision with water molecules. This is influenced by many biochemical and physical parameters [13]. Wolak et al. assessed drug diffusion ratios in the extracellular space of brain after intrathecal administration. They demonstrated that low molecular weight molecules, with a small hydrodynamic diameter and absence of fixation to negatively charged heparan sulfates, widely diffused in the extracellular space, reaching deep brain structures [13].

### 2.3. Clearance of the Cerebral Interstitial Fluid

Patlak et al. studied the efflux of radio-iodinated albumin after injection into the caudate nucleus, the internal capsule and the midbrain of rats [26]. After distribution in the interstitial fluid from the perivascular spaces, 62% of cerebral interstitial fluid drains into the CSF. Pharmacokinetic studies have demonstrated that tracers were cleared from the subarachnoid space: (i) to the blood through the arachnoid villi or fenestrated vessels of the choroid plexus [15,26,27], (ii) to the retropharyngeal lymph nodes through the cribriform plate or the sheath of olfactory nerves [28].

Using Tie2-GFP:NG2-DsRed double reporter mice that enable arteries and veins to be distinguished on tissue sections, other authors have observed that tracers accumulate within the perivascular spaces of capillaries and parenchymal venules one hour after intracisternal injections [17]. The tracers then exited the brain via para-venous routes of the medial internal cerebral veins and the lateral-ventral caudal rhinal veins. These results demonstrate that interstitial cerebral fluid and CSF circulate and are cleared via the same para-arterial and para-venous spaces in the glymphatic system.

Finally, the glymphatic system plays a role in xenobiotic distribution in the brain after intracerebral or intrathecal administration. It also enables the elimination of molecules that are too large to cross the BBB or that lack specific receptors [29]. This convective system is closely regulated by the aquaporin 4 channels of astrocytic endfeet.

## 3. Efflux Systems and Drug Clearance

As the BBB restricts large hydrophilic molecules from entering the brain parenchyma, direct injection of xenobiotics into the cerebrospinal fluid is a promising method to bypass the lack of permeability of the blood–brain barrier [30,31]. However, this approach has limitations, since drug transporters are responsible for an active efflux of xenobiotics from brain to blood. These transporters, mainly ATP-binding cassette (ABC) transporters and neonatal Fc receptor (FcRn), are expressed by endothelial cells in the BBB. This efflux, by preventing drugs from reaching relevant concentrations in the brain parenchyma, could explain their limited efficacy after intravenous administration and also after intracerebrospinal fluid administration [32,33,34,35,36,37].

### 3.1. ABC Transporters 

**Figure 2:**
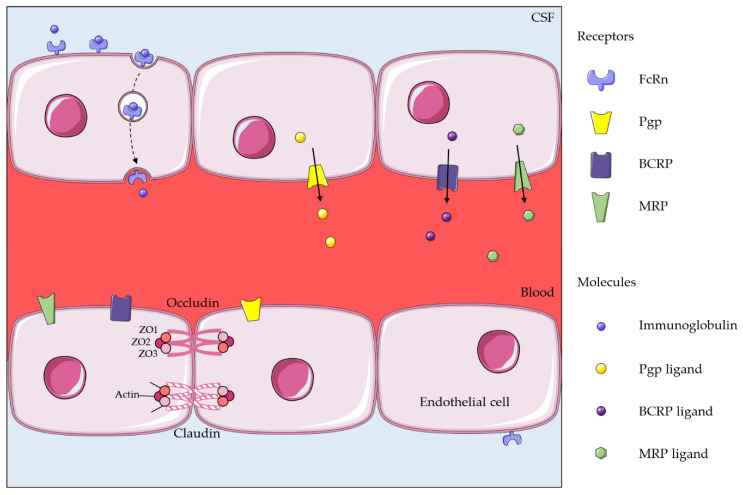
Efflux systems on the BBB (blood–brain barrier). Left side: transcytosis across endothelial cells of the BBB by FcRn. Middle and right side: ABC transporters at the luminal side of endothelial cells of the BBB. ZO: zonula occludens. (Images modified from © SMART/CC-BY-3.0).

The *ABC* gene family codes for 48 ABC transporters, which use energy from ATP hydrolysis to transport substrates across biological membranes, and six of them are implicated in drug transport [38]. For the BBB, the most widely studied ABC efflux transporters are the P-glycoprotein (Pgp), the breast cancer resistance protein (BCRP) and the multidrug resistance protein (MRP) [39] (Figure 2).

In humans, the Pgp is a 170 kDa plasma membrane protein of the ABC subfamily B member 1. It is encoded by two members of the Pgp gene family, *MRD1* and *MRD3.* In *Mus musculus*, Pgp is encoded by three genes, *mrd1*, *mrd2* and *mrd3* [40]. Only MRD1 proteins in humans, and mrd1 and mrd3 proteins in *Mus musculus*, enable multidrug transport through the BBB [41,42,43,44].

Pgp was identified in 1976 in Chinese hamster ovary cells [45]. In 1987, on a panel of eight mammalian cell lines, expression of the Pgp protein was associated with a multidrug resistance phenotype towards 15 anticancer drugs [46]. In human tissues obtained after surgery or autopsy procedures, following immunostaining, Pgp was mainly expressed by liver cells, renal tubule cells and gut endothelial cells, suggesting its role in the physiological elimination of xenobiotics [47,48]. In the human brain, Pgp was strongly expressed in the apical membrane of capillary endothelial cells [49,50,51], and also by astrocyte endfeet covering endothelial cells in the BBB [52]. This physiological expression at the BBB suggests a protective role of Pgp, promoting xenobiotic efflux from brain to blood. Even if Pgp shares the same function across species, its expression level at the BBB seems to be species dependent, and 3-times higher in human brain samples obtained from autopsy than in brain tissue obtained from Sprague Dawley laboratory rats [53]. Knockout mice lacking the functional homologue of human MDR1 protein have an abnormal accumulation of drugs in the brain parenchyma [54]. Surprisingly, when in vitro cell lines are exposed to increasing concentrations of a drug, the expression level of Pgp also increases [55,56]. This could be explained by the fact that Pgp expression, in addition to its physiological expression, is also inducible. More recently, in vitro and in vivo preclinical studies have evidenced drug-inducible expression of Pgp in response to exposure to certain xenobiotics [57,58].

In cancer patients with brain metastases or primary brain tumours, Pgp expression at the BBB is challenging clinical practice, since Pgp recognizes various cancer drugs (Table 1). To favour BBB passage and prevent drug efflux linked to Pgp expression, Pgp inhibitors have been tested in preclinical models, with promising results [59,60]. The aim was to increase drug accumulation in the brain parenchyma, including anticancer drugs (Table 2). Typically for taxanes (paclitaxel or docetaxel), combining their administration with Pgp inhibitor increases their brain parenchyma penetration 2- to 6-fold. However, it appears that Pgp inhibitors are less effective in inhibiting Pgp at the BBB than in other localizations [61], so that the use of higher doses is required, thus possibly entailing greater toxicity. In humans, the data are still limited. In healthy volunteers, a pilot study has shown that oral administration of tariquidar, a third generation Pgp inhibitor, increases the brain distribution volume of (R)-^11^C-verapamil by 24% compared to (R)-^11^C-verapamil only [62].

Finally, an additional complexity is linked to the possible activation of Pgp activity through conformational change induction, as shown with oxygenated xanthones [63,64].

BCRP is predominantly expressed in the luminal membrane of BBB endothelial cells [65,66]. It is implicated in drug resistance to several tyrosine kinase inhibitors, such as imatinib and gefitinib [67,68,69]. BRCP knockout mice showed an increased brain penetration of xenobiotics [70,71,72,73,74]. Even if Pgp is the main efflux transporter [75], BCRP and Pgp concomitantly act as efflux transporters, BCRP being more rapidly saturated than Pgp [76]. BCRP and Pgp have compensatory systems and probably need to be inhibited simultaneously to increase the brain distribution of drugs.

MRP is ubiquitously expressed in several tissues including the luminal membrane of BBB endothelial cells [77,78,79] and it acts as an anion transporter and also as a drug transporter [80]. Indeed, several drugs are both substrates for Pgp and MRP transporters (Table 1) as they have a synergistic and overlapping role in reducing the entrance of xenobiotics into the brain [81].

### 3.2. FcRn

FcRn, a heterodimer belonging to the major histocompatibility class I complex, is physiologically and ubiquitously expressed in humans, particularly in placental endothelial cells and in epithelial cells from the gut [108,109]. FcRn enables IgG and albumin to escape endothelial catabolism and transcytosis, thus promoting their biodistribution in the body. In particular, FcRn receptor enables fetal immunity through transfer of maternal immunoglobulins across the placenta [110,111].

In 2002, FcRn receptor expression was demonstrated for the first time in microvascular endothelial cells from rat brain using immunochemistry [112]. FcRn is expressed in the abluminal side of the endothelial cells of the BBB, and is involved in the transcytosis of immunoglobulins from brain to blood [113]. In a rat model, intracranial administration of two variants of a recombinant human immunoglobulin G1, one with high FcRn affinity and the other with low FcRn affinity, led to a high brain-to-blood efflux of the high FcRn affinity immunoglobulins [113]. Once the Fc fragment of the immunoglobulin binds to the FcRn receptor, an endocytosis process allows the immunoglobulin to enter the cytoplasm of the endothelial cell, and to be further expelled to the luminal side by an exocytosis process, as shown in vitro using fluorescent imaging [114,115] (Figure 2). Saturation can be reached after addition of the Fc fragment but not the Fab fragment of immunoglobulins [33].

In cancer patients, several approaches are being explored to improve brain penetration of drugs. Two methods are being developed to overcome the blood–brain barrier: (i) disruption of the blood–brain barrier, including intrathecal administration [11,12,116], intra-arterial administration [2,117,118,119,120,121,122], mechanical disruption of the BBB using osmotherapy [123], radiation [124,125] or ultrasounds [126]; (ii) methods without disruption of the BBB, for instance nano functionalization of drugs to cross the BBB or intranasal administration [2,127]. To date, most clinical approaches use the intrathecal route with a lumbar or an Ommaya reservoir, since direct intrathecal injections of drugs have led to some clinical benefit for the treatment of carcinomatosis meningitidis and parenchymal brain metastases [11,12,128,129]. This approach is simple and safe in daily practice but requires strict asepsis [130].

Even if these innovative approaches enable drugs to penetrate brain parenchyma, efflux transporters prevent relevant pharmacological concentrations from being reached. This is particularly true for therapeutic monoclonal antibodies, probably via FcRn receptors. In a pilot clinical study, our research team demonstrated considerable and rapid efflux of trastuzumab, an anti-HER2 IgG1, from CSF to blood after intrathecal administration [129]. Injections three times a week were required to reach relevant trastuzumab concentrations in the CSF, providing clinical efficacy in halting brain metastasis progression. Engineering therapeutic Fab fragments could be a way to overcome this limitation.

## 4. Conclusions

Brain metastases remain a major therapeutic challenge, since standard treatments have limited efficacy. Since primary tumors and metastases, including brain metastatic localizations, frequently share common genomic signatures [2], we assume a similar theoretical efficacy of targeted therapies on brain and extracentral nervous system metastases on the condition that there is sufficient pharmacological exposure. As a result, the same drug screening methods can be used, especially pre-clinical in vivo models of patient-derived xenografts, particularly relevant tools for the treatment of metastatic cancers [131]. It is however interesting to note that orthotopic patient-derived xenografts of brain metastases encounter the same problems of low permeability of the blood–brain barrier to drugs after intravenous injection, and that their use for the preclinical development of drugs should probably involve the intrathecal route of drug administration.

The intrathecal route has been proposed to facilitate drug access to the brain parenchyma via the glymphatic system. Diffusion into deeper brain areas depends on the molecular weight and the biochemical characteristics of drugs, and also on efflux systems from the BBB. Further studies are required to overcome these limitations and efficiently treat cancer brain metastases.

## Figures and Tables

**Table 1:** ABC transporter substrates.

ABC Transporters (*Corresponding Gene*)	Substrates	Biological Effect	Reference
**Pgp** ***(ABCB1)***	Anticancer drug		
	Paclitaxel	Microtubule polymer stabilizer	[82,83]
	Docetaxel	Microtubule-disruptive drug	[83,84]
	Vinblastine	Microtubule-disruptive drug	[83]
	Vincristine	Topoisomerase II inhibitor	[83]
	Mitoxantrone	Topoisomerase II inhibitor	[83]
	Etoposide (VP16)	Topoisomerase II inhibitor	[83]
	Doxorubicin	DNA intercalating agent	[83]
	Daunorubicin	DNA intercalating agent	[83]
	Afatinib	EGFR-TKI	[85]
	Erlotinib	EGFR-TKI	[85,86]
	Gefitinib	EGFR-TKI	[85,86]
	Osimertinib	EGFR-TKI	[85,86]
	Rociletinib	EGFR-TKI	[85]
	Anti-arrhythmic agent		
	Digoxin	Sodium pump inhibitor	[87]
	Anti-retroviral drug		
	Ritonavir	Protease inhibitor	[88]
	Saquinavir	Protease inhibitor	[88]
	Anti-inflammatory		
	Colchicine	Microtubule assembly inhibitor	[89]
**MRP** ***(ABCC1)***	Anticancer drug		
	Etoposide (VP16)	Topoisomerase II inhibitor	[83]
	Anti-inflammatory		
	Colchicine	Microtubule assembly inhibitor	[89]
**MRP** ***(ABCC2)***	Anticancer drug		
	Vinblastine	Microtubule-disruptive drug	[83,90]
	Uricosuric drug		
	Sulfinpyrazone	Degranulation of platelets inhibitor	[90]
**MRP** ***(ABCC3)***	Anticancer drug		
	Topotecan	Topoisomerase I inhibitor	[83]
	Camptotecin (CPT-11)	Topoisomerase I inhibitor	[91]
	Etoposide (VP16)	Topoisomerase II inhibitor	[83]
	Mitoxantrone	Topoisomerase II inhibitor	[83]
	Doxorubicin	DNA intercalating agent	[83]
	Daunorubicin	DNA intercalating agent	[83]
	Methotrexate	Dihydrofolate reductase inhibitor	[92]

EGFR-TKI: Epidermal Growth Factor Receptor Tyrosine Kinase Inhibitor.

**Table 2:** Brain exposure to xenobiotics combined with Pgp inhibitors in preclinical studies.

Pgp Inhibitor (*Mechanism of Action*)	Dose	Time Lapse before Drug Administration	Drug Tested	Dose	Time Lapse before Brain Analysis	Increased Brain Parenchyma Penetration	Species	Reference
Cyclosporin A *(Calcineurin inhibitor)*			Anticancer drug					
	50 mg/kg p.o	1 h	Paclitaxel	10 mg/kg i.v	24 h	3 times	Mice	[93]
	50 mg/kg p.o	1 h	Docetaxel	33 mg/kg i.v	24 h	2.3 times	Mice	[94]
			Antidepressant					
	20 mg/kg i.p	1 h	Escitalopram	0.1 mg/kg i.p	30 min	>2 times	Mice	[95]
	20 mg/kg i.p	1 h	Escitalopram	1 mg/kg i.p	30 min	>1.75 times	Mice	[95]
	200 mg/kg i.p	1 h	Nortriptyline	10 mg/kg i.p	1 h	1.5 times	Rats	[96]
	25 mg/kg i.v	30 min	Imipramine	5 mg/kg i.v	4 h	1.84 times	Rats	[97]
			Opioid					
	100 mg/kg i.p	1 h	Oxycodone	1 mg/kg s.c	2 h	1.4 times	Mice	[98]
Zosuquidar *(MDR1 inhibitor)*			Anticancer drug					
	25–80 mg/kg p.o	1 h	Paclitaxel	10 mg/kg i.v	24 h	2.1–5.6 times	Mice	[99]
	25 mg/kg i.p	30 min	Imatinib	25 mg/kg p.o	1 h	2–3 times	Mice	[100]
Elacridar *(MDR1 and BRCP inhibitor)*			Anticancer drug					
	25 mg/kg p.o	2 h	Paclitaxel	10 mg/kg i.v	24 h	5 times	Mice	[94]
	25 mg/kg p.o	2 h	Docetaxel	33 mg/kg i.v	24 h	3.6 times	Mice	[93]
	100 mg/kg p.o	2 h	Sunitinib	10 mg/kg p.o	1 h	12 times	Mice	[101]
	100 mg/kg p.o	15 min	N-desethyl sunitinib	5 mg/kg i.v	1 h	3.3 times	Mice	[102]
	5 mg/kg i.p	30 min	Lapatinib	100 mg/kg p.o	24 h	1.5 times	Rats	[103]
	100 mg/kg p.o	2 h 30 min	Vemurafenib	5 mg/kg p.o	4 h	3–5 times	Mice	[104]
	100 mg/kg p.o	2 h	Crizotinib	5 mg/kg p.o	4 h	2.2 times	Mice	[105]
	10 mg/kg i.v	30 min	Gefitinib	25 mg/kg p.o	2 h	4 times	Mice	[106]
Valspodar *(MDR1 inhibitor)*			Anticancer drug					
	25 mg/kg p.o	1 h	Paclitaxel	10 mg/kg i.v	24 h	6.5 times	Mice	[93]
	25 mg/kg p.o	1 h	Docetaxel	33 mg/kg i.v	24 h	3.5 times	Mice	[94]
	10 mg/kg i.v	5 min	Vinblastine	brain perfusion	20 s	9.1 times	Rats	[107]
			Anti-inflammatory					
	10 mg/kg i.v	5 min	Colchicine	brain perfusion	20 s	8.4 times	Rats	[107]
Verapamil *(Calcium channel inhibitor)*			Anticancer drug					
	1 mg/kg i.v	5 min	Vinblastine	brain perfusion	20 s	3.7 times	Rats	[107]
			Anti-inflammatory					
	1 mg/kg i.v	5 min	Colchicine	brain perfusion	20 s	3.7 times	Rats	[107]
			Antidepressant					
	20 mg/kg i.p	1 h 30 min	Imipramine	5 mg/kg i.v	4 h	1.44 times	Rats	[97]
			Opioid					
	3 mg/kg i.p	1 h	Oxycodone	1 mg/kg s.c	2 h	1.3 times	Mice	[98]

per os (p.o); intravenous (i.v); intraperitoneal (i.p); subcutaneous (s.c).

## Abbreviations

ABC	ATP-Binding Cassette
BBB	Blood–Brain Barrier
BCRP	Breast Cancer Resistance Protein
CSF	Cerebrospinal Fluid
FcRn	Neonatal Fc Receptor
MRD	Multidrug Resistance Protein
Pgp	P-Glycoprotein
